# Detection of Pulmonary Nodules in CT Images Based on Fuzzy Integrated Active Contour Model and Hybrid Parametric Mixture Model

**DOI:** 10.1155/2013/515386

**Published:** 2013-04-16

**Authors:** Bin Li, Kan Chen, Lianfang Tian, Yao Yeboah, Shanxing Ou

**Affiliations:** ^1^School of Automation Science and Engineering, South China University of Technology, Guangdong, Guangzhou 510640, China; ^2^Department of Radiology, Guangzhou General Hospital of Guangzhou Command, Guangdong, Guangzhou 510010, China

## Abstract

The segmentation and detection of various types of nodules in a Computer-aided detection (CAD) system present various challenges, especially when (1) the nodule is connected to a vessel and they have very similar intensities; (2) the nodule with ground-glass opacity (GGO) characteristic possesses typical weak edges and intensity inhomogeneity, and hence it is difficult to define the boundaries. Traditional segmentation methods may cause problems of boundary leakage and “weak” local minima. This paper deals with the above mentioned problems. An improved detection method which combines a fuzzy integrated active contour model (FIACM)-based segmentation method, a segmentation refinement method based on Parametric Mixture Model (PMM) of juxta-vascular nodules, and a knowledge-based C-SVM (Cost-sensitive Support Vector Machines) classifier, is proposed for detecting various types of pulmonary nodules in computerized tomography (CT) images. Our approach has several novel aspects: (1) In the proposed FIACM model, edge and local region information is incorporated. The fuzzy energy is used as the motivation power for the evolution of the active contour. (2) A hybrid PMM Model of juxta-vascular nodules combining appearance and geometric information is constructed for segmentation refinement of juxta-vascular nodules. Experimental results of detection for pulmonary nodules show desirable performances of the proposed method.

## 1. Introduction

Pulmonary nodules in high-resolution CT images are potential manifestations of lung cancer. However, the interpretation of a great deal of CT images brings a huge workload upon the radiologist, which in turn increases the false negative rate due to observational oversights. CAD system for pulmonary nodules plays an important role in the diagnosis of lung cancer [1], which assists doctors in the interpretation of medical CT images and increases the detection of lung cancer by reducing the false negative rate as a result of observational oversights. Detection of different types of nodules in a CAD system is a difficult task. As pointed by the literatures [2, 3], despite much effort being devoted to the computer-aided nodule detection problem, CAD for various types of pulmonary nodules remains an ongoing research topic. One of the major difficulties is the task of detecting nonsolid and part-solid GGO nodules with faint contrast and fuzzy margins. In particular, nonsolid nodules are extremely subtle with fuzzy boundaries, and part-solid nodules exhibit highly irregular intensity variations (intensity inhomogeneity) and boundary shapes. Studies have shown that nodules of nonsolid and part-solid nature are frequent and have higher risks of being malignant than solid ones [2]. Additionally, there are difficulties associated with the detection of nodules, that are adjacent to vessels when they have very similar intensities, and the detection of nodules, that are nonspherical in shape. Moreover, juxtavascular nodules account for the largest typology of lung nodules [3]. Thus, handling them under a united framework poses a great challenge to the task of segmentation of pulmonary nodules. Although various algorithms have been reported in the literatures [2–5] for tackling these problems, many technical issues still remain, including accurate segmentation and detection. Furthermore, those that are applicable to various densities of GGO and juxtavascular nodules have not been available until recently. In such cases, traditional segmentation methods may lead to boundary leakage and also result in “weak” local minima. Additionally, purely intensity thresholding or model-based detection methods may fail to identify GGO and juxtavascular nodules, and their detection error rate may be unacceptably high. All these factors lead to the belief that the field is relatively new and requires further investigation. This paper deals with the above-mentioned problems. In this paper, an improved detection method for pulmonary nodules in CT images, which combines the FIACM-based segmentation, PMM-based segmentation refinement of juxtavascular nodules, and knowledge-based classifier, is proposed for detecting various types of pulmonary nodules, especially for GGO nodules (part-solid and nonsolid) and juxtavascular nodules.

### 1.1. Previous Work on Detection of Pulmonary Nodules

In CAD of pulmonary nodules, segmentation of potential nodule objects is the first necessary and crucial step. In the segmentation step, the potential nodule objects for training and testing data sets of classification are generated. However, the segmentation of GGO nodules and juxtavascular nodules is a very difficult task. It is difficult to acquire an ideal segmentation effect only by relying on general image-data-driven segmentation methods. Active contour models have been one of the most successful methods for image segmentation [6, 7], hence their implementation in the segmentation of pulmonary nodules [8]. However, for nonsolid/part-solid GGO nodules or juxtavascular nodules in real-world image, typical weak edges as well as intensity inhomogeneities may exist and hence boundaries are difficult to define. In such cases, the purely edge-based active contour models [9, 10], which rely on edge functionality to terminate the curve evolution for detecting objects with edges defined by gradient, are likely to yield undesirable local minima, so their performance is often inadequate. In addition, purely region-based active contour models [6, 7, 11] may be more sensitive to noise and cannot handle objects with ill-defined boundaries, hence may also cause problems of boundary leakage and “weak” local minima. To overcome the limitations of traditional region-based active contour model, Krinidis and Chatzis [12] used the fuzzy energy to provide a balanced technique with a strong ability to reject “weak” local minima. van Assen et al. [13] proposed a 3D active shape model driven by fuzzy inference (application to cardiac CT and MR). Li et al. [7] proposed a region-based active contour model incorporating a data fitting energy to overcome the difficulties caused by intensity inhomogeneities. Furthermore, there has been much research into the design of complex integrated active contour model combining edge and region energy [14] in order to overcome the limitations of traditional active contour models. 

Besides handling nonsolid or part-solid GGO nodules, it is also important for a segmentation algorithm to be able to treat juxtavascular nodules. Juxtavascular nodules account for the largest typology of lung nodules [3]. Thus, handling them under a united framework presents a great challenge to the segmentation task of GGO and juxtavascular nodules. Different approaches have been proposed to outline lung nodules close to vessels. Morphological operators were largely investigated for the separation of the nodule from blood vessels [3, 15, 16]. However, the sizes and shapes of vessels as well as those of nodules are irregular; hence it is very difficult to obtain an acceptable segmentation result if only morphological correction is relied upon. For example, Kostis et al. performed removal of vessels by means of a morphological operator having a constant size [15], which may lead to the problem of a small volume overestimation at the vessel attachment. Kuhnigk et al. [16] carried out a more complex morphological correction, supposing that the size of vessels decreases while the vessels evolve along the periphery of the lungs. However, this is not always the case, especially like those in vessel branches; hence the performance is inadequate. The methods for detachment of vessels from segmented nodules usually employ strategies which involve the entire nodule boundary with the possible drawback of making the segmentation worse where no attachment occurs. Hence, a better segmentation refinement method should be taken into consideration further. In addition, some literatures have aimed at building some statistical models for pulmonary nodules and blood vessels [17–19]. However, approaches utilizing simple criteria like shape rule or gray value evidence are typically not suitable to differentiate between different tubular tree structures and nodules. Models, in a broad sense, are embedded prior information about the target structures [20, 21]. To our knowledge, no hybrid models combining appearance and geometric information by incorporating assumptions on the spatial appearance of a vessel and its attached pulmonary nodules have been presented and discussed. Also present statistical models have not included both of the local intensity and structure features. 

After the potential nodule objects are segmented exactly, every potential nodule object is evaluated and classified individually for the probability of true positive. There are a number of classification techniques used in the stage of the nodule detection CAD systems: rule-based or linear classifier [22–24], neural network [25], multilayer perception, SVM (support vector machine) [4], and so on. For example, Hardie et al. [26] also recently proposed a CAD system for identifying lung nodules in 2D chest radiographs that consists in using a weighted mean convergence index detector and an adaptive distance-based threshold algorithm to segment the detected nodule candidates. In the literature [26], a set of 114 features is computed for each candidate. This is followed by a classifier to reduce FPs. A Gaussian Bayes linear classifier, a Fisher linear discriminant (FLD) classifier, and a quadratic classifier are compared. Many other groups have also recently presented systems and performance studies for detecting nodules [27]. However, as for nonsolid/part-solid GGO nodules and juxtavascular nodules, the error rate of purely intensity or model-based detection methods may be much higher. For this reason, we believe that the field is relatively new and requires further investigation.

### 1.2. Our Approach

**Figure 1:**
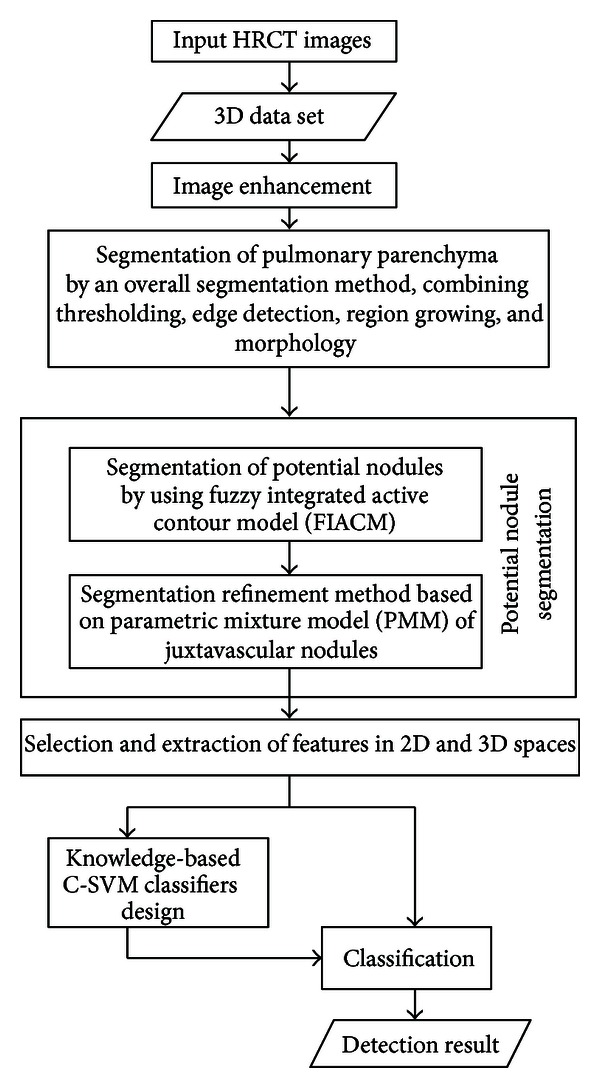
Overview of the proposed detection method for pulmonary nodules.

This paper deals with the above-mentioned problems. In this paper, an improved detection method of pulmonary nodules in chest CT images, combining the FIACM-based segmentation, PMM-based segmentation refinement for juxtavascular nodules, and a knowledge-based C-SVM classifier, is proposed for detecting various types of pulmonary nodules, especially for GGO nodules (part-solid and nonsolid) and juxtavascular nodules. The flowchart of the proposed detection algorithm for pulmonary nodules under a united framework is shown in Figure 1.

Compared with existing traditional methods, our approach has several novel aspects:

1. in order to overcome the problems of boundary leakage and “weak” local minima in segmentation of part-solid/nonsolid GGO and juxtavascular nodules using traditional segmentation methods, the paper proposes a fuzzy integrated active contour model (FIACM), in which edge and local region information is incorporated. A new edge-stopping function is specified based on posterior probability. The statistical information of local region in a dynamic mask, combining the fuzzy energy, is introduced into the active contour energy function model, and the fuzzy energy is used as the model motivation power for the evolution of the active contour.
2. To overcome the problem of a small volume overestimation at the vessel attachment and get a good segmentation result of juxtavascular nodules under a united segmentation framework, a hybrid PMM of juxtavascular nodules combining appearance and geometric information is constructed for segmentation refinement of juxtavascular nodules.
3. A knowledge-based C-SVM classifier is constructed, using some 2D and 3D features.

The remainder of this paper is organized as follows. In Sections 2 and 3, the proposed segmentation methods of potential nodule objects are first introduced. Then, the proposed classification algorithm based on knowledge-based C-SVM classifier is presented in Section 4. Finally, the experimental results of our method are given in Section 5, followed by some discussions in Section 6. This paper is summarized in Section 7.

## 2. Fuzzy Integrated Active Contour Model-Based Segmentation 

### 2.1. The Proposed Fuzzy Integrated Active Contour Model (FIACM)

 Let *Ω* ⊂ *R* ^3^ be the image domain, and let *D* : *Ω* → *R* be the given medical CT image sequence or 3D data set. The segmentation result of the images or data set (for 3D data set) *D* is achieved by finding a surface *ϕ*, which separates *Ω* into disjoint regions. *Ω*_1_ and *Ω*_2_ represent the inside regions and outside regions of *ϕ*, respectively. Besides intensity, more features are used in our active contour model. Taking both the edge and local region information into consideration, our proposed energy function model is given as follows:

(1) E(f1,f2,ϕ) =μ∫ϕδϕ(p)gcolor|∇ϕ|dp  +∫Ω1λ1[u(p)]m(I(p)−f1(x))2Hϕ(p)dp  +∫Ω2λ2[1−u(p)]m(I(p)−f2(x))2(1−Hϕ(p))dp,

where *E*(*f*_1_, *f*_2_, *ϕ*) is the proposed energy function model; *f*_1_(x) and *f*_2_(x) are two values that approximate image intensities in the local regions *Ω*_1_ and *Ω*_2_, respectively. x(*x*, *y*, *z*) ∈ *Ω* is a given pixel/voxel, also a location variable.

On the right-hand side of (1), *p*(*x*, *y*, *z*) ∈ *Ω* is a location variable like x; *I*(*p*) represents the intensity value in *p*; the first term *μ*∫_*ϕ*_ *δϕ*(*p*)*g*_color_|∇*ϕ*|*dp* is the edge-driven energy term, which is used for curved surface to improve the evolved ability in concave regions; *μ* is the weight of edge-driven energy term; *δϕ*(*p*) is the smoothed version of the Dirac delta; *g* is the stop function. The second and third terms are the region-driven energy terms, which are used to control the image force based on statistical region-intensity information; *λ*_1_ and *λ*_2_ are the weights for region-driven energy term. The membership function *u*(*p*) ∈ [0, 1] is the degree of membership of *D*, and *m* is a weighting exponent on each fuzzy membership. The degree of membership is decided by not only the intensity feature, but also the shape feature: local shape index. *Hϕ* is the smoothed Heaviside function.

The proposed FIACM model differs from the model used in the literatures [7, 12–14], which has several novel aspects. (1) the right-hand side of (1) consists of the edge-driven energy term and region-driven energy terms. The edge and local region information is incorporated into the proposed FIACM model. (2) In the second and third terms of right-hand side of (1), the statistical information of local region in a dynamic mask, combining the fuzzy energy, is introduced into the active contour energy function model. This will be explained in detail below in Section 2.2. (3) The fuzzy energy is used as the model motivation power evolving the active contour, which is represented and communicated by the membership function *u*(*p*) in the second and third terms of right-hand side of (1). The degree of membership is decided by not only the intensity feature, but also the shape feature: local shape index. So more features, including intensity and local shape index, are used in the proposed active contour model. This will be explained in detail below in Section 2.3. (4) In the first term of right-hand side of (1), a new edge-stopping function *g* is specified based on posterior probability. This will be explained in detail below in Section 2.4.

In the simple case, it is obvious that the boundary of the object *ϕ*_0_ is the minimizer of the energy functional. The energy function model as (1) is solved by using variational level set approach, and by taking the first variation of this energy with respect to *ϕ* the following evolution equation is obtained follows:

(2) ∂ϕ∂t=δ∈{μdiv⁡(gcolor∇ϕ|∇ϕ|)   −∑i=1kλ1(i)[u(p)]m(I(p)−f1(i)(x))2   +∑i=1kλ2(i)[1−u(p)]m(I(p)−f2(i)(x))2}.

### 2.2. Local Region-Scalable Flexible Fitting Energy in FIACM Model

In order to improve the capability of handling intensity inhomogeneity and objects with ill-defined boundaries, while reducing the computation cost, the statistical information of a local region is considered in the active contour energy function model. We utilize a local intensity fitting energy as the region-scalable fitting energy. In our energy function model as in (1), *f*_1_(x) and *f*_2_(x) are flexible fitting values, which are two values that approximate image intensities in the local region decided by a flexible mask of *B*(*k*). That is, the intensities *I*(*p*) that are effectively involved in the above fitting energy are in a local region centered at the point x, whose size can be controlled by the mask *B*(*k*). 

A 3D mask, *B*(*k*) as described by (3), possesses statistical information of the local region. It is a characteristic function and is introduced into the energy function:

(3) $$\begin{array}{c} B\left(k\right)=\{\begin{array}{cc} \frac{1}{{\left(2\pi \right)}^{n/2}\sigma^{n}}e^{-{\left|k\right|}^{2}/2\sigma^{2}}, & k\in \left\{\Omega_{1}\cup \Omega_{2}\right\}\\ 0, & \text{otherwise}. \end{array} \end{array}$$

The kernel function, *B*(*k*), with a scale parameter *σ*, is controlled by the mean of the degrees of membership in the region. In our proposed model as illustrated in (1), a window will be defined; so *k* = x − *p*. 

Therefore, the local intensity fitting energy in (3) is called as the region-scalable fitting (RSF) energy of a contour *C* at a point x. Using the statistical information of local region, the means *f*_1_(x) and *f*_2_(x) in the interior region *Ω*_1_ and exterior region *Ω*_2_ can be computed from (4). Therefore, the evolution of the point in the image domain is only related to the intensities in surrounding region, while it is independent of the region beyond the mask field. Hence, the difficulties caused by intensity inhomogeneities are overcome:

(4) $$\begin{array}{c} f_{1}\left(x\right)=\frac{B\left(x-p\right)*\left[H\phi \left(x\right)I\left(x\right)\right]}{B\left(x-p\right)*H\phi \left(x\right)},\\ f_{2}\left(x\right)=\frac{B\left(x-p\right)*\left[\left(1-H\phi \left(x\right)\right)I\left(x\right)\right]}{B\left(x-p\right)*\left(1-H\phi \left(x\right)\right)}. \end{array}$$

### 2.3. The Degree of Membership in FIACM Model

**Figure 2:**
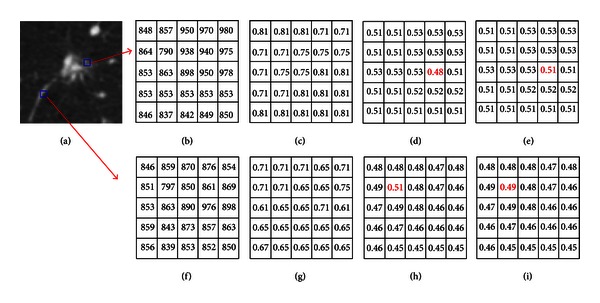
Intensity values, shape index values, and the degree of membership of a GGO juxtavascular pulmonary nodule and its attached vessel. (a) Original CT image; (b) intensity of juxtavascular nodule; (c) shape index values of juxtavascular nodule; (d) the degree of membership of vessel before the fuzzy morphological filtering; (e) the degree of membership of juxtavascular nodule; (f) intensity of vessel; (g) shape index values of vessel; (h) the degree of membership of vessel before the fuzzy morphological filtering; and (i) the degree of membership of vessel after the fuzzy morphological filtering.

In our FIACM model, the fuzzy energy is used as the model motivation power evolving the active contour. As shown in (1), *u*(*p*) : *X* → [0, 1] defines the membership degree of a pixel *p* in data set *D* to the nodule class cluster center. The corresponding degree of membership of each pixel in the data set should be calculated. Thus, the degree of membership for each sample *X*_*i*_ = (*x*_*i*_, *y*_*i*_, SI_*i*_, *I*_*i*_) in our FIACM model is calculated by using the fuzzy clustering algorithm based on intensity and local shape index. Meanwhile, the fuzzy morphological opening operation [28] is used to eliminate noise. Here, in the clustering space, a sample is *X*_*i*_ = (*x*_*i*_, *y*_*i*_, SI_*i*_, *I*_*i*_); *x*_*i*_ and *y*_*i*_ is the position feature; *I*_*i*_ is the intensity feature and *SI*_*i*_ is the local volumetric shape index. The volumetric shape index (SI) [4] is a measure of local shape characteristics. The local shape index SI(*p*) at pixel *p* can be defined by

(5) $$\begin{array}{c} \text{SI}\left(p\right)=\frac{1}{2}-\frac{1}{\pi }\arctan\frac{k_{1}\left(p\right)+k_{2}\left(p\right)}{k_{1}\left(p\right)-k_{2}\left(p\right)}, \end{array}$$

where *k*_1_(*p*) and *k*_2_(*p*) are principal curvatures at pixel *p*. This index can be used to distinguish spherical from cylindrical shapes: values close to 1 indicate spherical shapes, while values close to 0.75 indicate cylindrical shapes. However, pulmonary nodules are hard to distinguish if merely the intensity or the shape index features are utilized. Therefore, the degree of membership is an important feature for segmentation or even the detection of GGO or juxtavascular pulmonary nodules. Generally speaking, the degree of membership of pulmonary nodules is greater than 0.5, and the degree of membership of blood vessel is less than 0.5. An example is illustrated in Figure 2.

The intensity values, shape index values, and the degree of membership values of a GGO juxtavascular pulmonary nodule and its attached blood vessel are shown in Figure 2. As shown in Figures 2(b), 2(c), 2(f), and 2(g), pulmonary nodules are hard to distinguish if only intensity or the shape index features are relied upon. As shown in Figures 2(d) and 2(h), some noise, indicated in red, occurs when the data are transformed into the fuzzy domain. The fuzzy morphological filtering is, therefore, adopted to eliminate the noise. As shown in Figures 2(e) and 2(i), the degree of membership of pulmonary nodules is greater than 0.5, and the degree of membership of blood vessel is less than 0.5. This implies that the degree of membership is an important feature for segmentation or even detection of GGO or juxtavascular nodule.

### 2.4. Selection of Edge-Stopping Function Based on Posterior Probability

In the proposed FIACM model, a new edge-stopping function is specified based on posterior probability. The edge-stopping function is important and has a strong impact on the final outcome of curved surface evolution. As for nonsolid or part-solid GGO pulmonary nodules with faint contrast and fuzzy margins, rough and weak edges or even concave edges are often exhibited, and their regions often possess intensity inhomogeneities. Since real images do not contain ideal edges, an edge-stopping function, *g*, must be specified. The main goal of stopping function, *g*, is actually to stop the evolving curved surface when it arrives at the objects boundaries.

**Figure 3:**
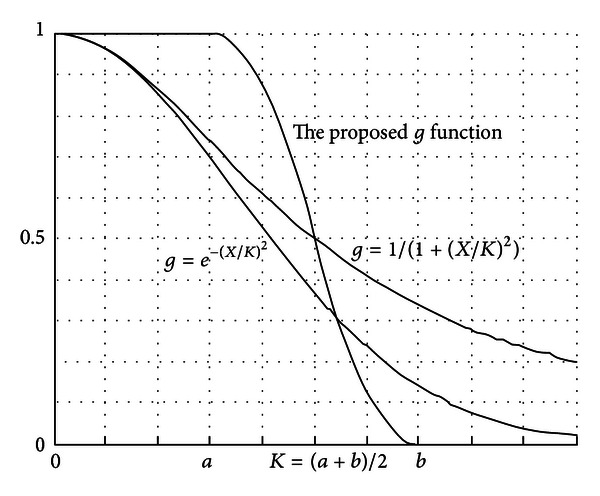
The specified edge-stopping function.

The edge-stopping function, *g*, is a nonlinear strictly increasing function, which is like the weather vane of convergence of curved surface evolvement. In the active contour model based on curvature diffusion, the edge-stopping function is often selected as *g*(∇*I*_*σ*_) = 1/(1 + (|∇*I*_*σ*_|/*K*)^2^) or *g*(∇*I*_*σ*_) = *e* ^−(|∇*I*_*σ*_|/*K*)^2^^(where ∇*I*_*σ*_ = ∇*I*∗*G*_*σ*_ is Gaussian gradient), whose function curves are shown in Figure 3. These two functions are similar to the Butterworth or Gaussian high-pass filter response function. As in the frequency domain analysis, the image edge corresponds to the high-frequency signals; hence these two functions can produce a strong response to the edge region. However, these two functions are also relatively very sensitive to noise; therefore, false edges are bound to be formed. Furthermore, they have a difficulty in handling concavities within the boundary. In this case, it is necessary to select an appropriate stopping function in our model. The specified new edge stopping function, illustrated in (6), is shown in Figure 3. The edge-stopping function we selected can be controlled by two gradient module thresholds *a* and *b*. The thresholds *a* and *b* are determined using posterior probability, and the conditional probability density function is defined as a Gaussian function of intensity and gradient, whose parameters are calculated using the expectation maximization algorithm. When *t* ≤ *a*, *g* = 1, this represents the homogeneous region; when *t* ≥ *b*, *g* = 0, the edge region is represented. Essentially, the stopping function describes the changing process in transition region (between the homogeneous region and edge region) using a second-order nonlinear smooth function. The edge stop function quantitatively describes the weak boundaries of the image, which can enhance the robustness of noise region and improve the evolution performance in intensity inhomogeneities situations and concave edges:

(6) $$\begin{array}{c} g\left(t\right)=\{\begin{array}{cc} 1 & t\leq a\\ 1-2{\left(\frac{t-a}{b-a}\right)}^{2} & a<t\leq \frac{a+b}{2}\\ 2{\left(b-\frac{t}{b-a}\right)}^{2} & \frac{a+b}{2}<t\leq b\\ 0 & t>b. \end{array} \end{array}$$

### 2.5. Implementation of Potential Pulmonary Nodule Segmentation Based on FIACM Model

The implementation algorithm for the FIACM model is as follows.

1. Compute the degree of membership *u*(*p*) according to Section 2.3.
2. Initialize the contour of the FIACM model based on adaptive local threshold segmentation. In the level set method for the proposed FIACM model, selection of the initial contour *ϕ*_0_ has a different effect on the efficiency of the algorithm implementation. As the lung is essentially a bag of air in the body, it shows up as a dark region in CT scans. Pulmonary nodules are often calcified tissues. This contrast between pulmonary nodules and surrounding tissues forms the basis for the majority of the segmentation schemes. So in this paper, the segmentation result of adaptive local threshold segmentation method is used as the initial contour of the following hybrid level set model, which greatly reduces the number of iterations of curved surface evolution and ensures that the desired segmentation effect is achieved.
3. Compute the scale parameter *σ* of the kernel function, *B*(*k*), which is controlled by the mean of the degrees of membership in a region, for example, a circle. Then proceed to compute the region-scalable fitting energies *f*_1_(*p*) and *f*_2_(*p*).
4. Determine the thresholds *a* and *b* of the stop function term using posterior probability and then specify the stop function term.
5. Implement the numerical algorithm of the proposed FIACM model. In this paper, the proposed FIACM model given as (2) is implemented by an efficient numerical algorithm based on an additive operator splitting (AOS) and Thomas algorithm [29].

## 3. Segmentation Refinement of Juxtavascular Nodules Based on Hybrid PMM

Compared with existing ones, the proposed hybrid PMM-based segmentation of juxtavascular nodules has several distinct features that include the following.

1. Various types of pulmonary nodules, especially for GGO nodules (part-solid and nonsolid) and juxtavascular nodules, are segmented under a united segmentation framework. In order to overcome the problem of a small volume overestimation at the vessel attachment and obtain a good segmentation result of juxtavascular nodules under a single-segmentation framework, a hybrid PMM of juxtavascular nodules is constructed for segmentation refinement of juxtavascular nodules. The refinement procession is just used for the pixels in some regions containing the blood vessels and juxtavascular nodules; hence the correction and refinement methods have the advantage of local refinement of the nodule segmentation along recognized vessel attachments only, without modifying the nodule boundary elsewhere. Moreover, it has the potential to reduce the computation cost involved.
2. The hybrid model takes both the appearance and geometric information into consideration. The hybrid model combines appearance and geometric information by incorporating assumptions on the spatial appearance of a vessel.

### 3.1. The Proposed Hybrid PMM of Juxtavascular Nodules

#### 3.1.1. Observation Vector Generation in the Proposed Hybrid PMM of Juxtavascular Nodules

In the models of juxtavascular nodules, in a broad sense, prior information about the target structures is embedded. Because CT values of pulmonary nodules and that of blood vessels are almost uniform, it is difficult to estimate exactly the parameters and segment the blood vessels and the attached nodules if merely the intensity feature is utilized and a statistical model is built based merely on the intensity feature. Our proposed hybrid PMM model takes both the appearance and geometric information into consideration. That is, a set of observation vectors are defined and extracted to include the appearance and geometric information. Appearance information expresses prior knowledge on the luminance properties of the vascular structures and the attached nodules. Depending on the application in segmentation and detection of juxtavascular nodules, additional knowledge about the specific geometric shape might be incorporated.

Thus, we define an extended observation vector as

(7) $$\begin{array}{c} v=\left(I_{i},u_{i},o_{i}\right), \end{array}$$

where *v* is the observation vector in position (*x*, *y*); *I*_*i*_ is the intensity feature; *u*_*i*_ is the degree of membership, which is from the volumetric shape index SI_*i*_, reflecting the geometric shape; the membership function *u*(*p*)∈[0, 1] and *o*_*i*_ is the regularized flow direction vector.

#### 3.1.2. The Regularized Flow Direction Feature of Nodules Adjacent to Blood Vessels

As vessels are characterized by a tubular model, the 3D gradient vectors in a vessel can be used to extract a vector in the direction of the vessel by identifying a vector that is approximately orthogonal to the gradients in a local neighborhood [17–19]. Hence, the flow direction can be used as a feature vector to build a statisticial PMM for segmentation of blood vessels and attached nodules.

In this paper, the structure tensors are computed by directly using the gradient information of each voxel, which is different from the method given in the literature [19]. In the literature [19], a 3 × 3 correlation matrix *GG* ^*T*^ is essentially a structure tensor defined by the statistical information of arithmetic average in local region, which will weaken local features in each voxel to a certain degree. We assume that *T* is the structure tensor, whose eigenvalues are *λ*_1_, *λ*_2_, and *λ*_3_ (*λ*_1_ ≤ *λ*_2_ ≤ *λ*_3_). Let *e*_1_ be the unit length eigenvector belonging to the eigenvalue *λ*_1_. A vector pointing in flow direction is set as

(8) $$\begin{array}{c} l_{1}=\sqrt{\lambda_{3}}\cdot e_{1}. \end{array}$$

Since vectors in opposite directions indicate the same direction of a blood vessel, it is possible to invert some of the vectors so that the majority of the vectors of the same blood vessel have consistent orientation. This can be achieved in a simple way by inverting all vectors with an angle difference in the range of [0, *π*]. Let *e* be the unit vector in the flow angle *π*/2. The regularized flow direction vector *o*_*i*_ is

(9) $$\begin{array}{c} o_{i}=\{\begin{array}{cc} -l_{i} & \langle l_{i},e\rangle \in [-1,0)\\ l_{i} & \text{otherwise}. \end{array} \end{array}$$

#### 3.1.3. The Proposed Hybrid PMM of Juxtavascular Nodules

As mentioned in Section 3.1.1, our proposed hybrid PMM takes both the appearance and geometric information into consideration. The observations, *v* = (*I*_*i*_, *u*_*i*_, *o*_*i*_), as described in Section 3.1.1, include both the appearance and geometric information. According to medical knowledge of anatomy, blood vessels are similar to tubes, whose flow directions show a Gaussian distribution [17]; and the pulmonary nodules are similar to spheres. Furthermore, the intensity values of pulmonary nodules and those of blood vessels are almost uniform. So assuming that the mixture components are multivariate Gaussian distributions [18, 19, 30], and that there is a uniform noise component in the mixture, a statistical PMM for the blood vessel and juxta-nodule is obtained:

(10) $$\begin{array}{c} f\left(v\;|\;\theta \right)=\sum_{i=1}^{M-1}\alpha_{i}\phi \left(v\;|\;\mu_{i},\Sigma_{i}\right)+\alpha_{M}p_{M}\left(v\right), \end{array}$$

where *f*(*v* | *θ*) is the density of a parametric finite mixture model; *v* is the observation vector, *v* = (*I*_*i*_, *u*_*i*_, *o*_*i*_); *θ* = (*α*_1_,…, *α*_*M*_, *θ*_1_,…, *θ*_*M*_) is the vector of parameters; *θ*_*i*_ is composed of the elements of *μ*_*i*_; ∑_*i*_. *ϕ*(*v* | *μ*_*i*_, ∑_*i*_) is the density of a multivariate Gaussian random vector with mean *μ*_*i*_ and covariance matrix ∑_*i*_; *p*_*M*_(*v*) is a multivariate uniform density function, *p*_*M*_(*v*) = 1/*π*; *α*_*i*_ is the structure weight coefficient (the inner mixing proportion), ∑_*i*=1_^*M*^ *α*_*i*_ = 1. *μ* and *σ* are the mean and variance of the multivariate Gaussian distribution, respectively. Parametric models for nodules and vessels can be obtained from (9) when using *M* = 3.

### 3.2. Implementation of PMM-Based Segmentation Refinement of Juxtavascular Nodules

The algorithm for PMM-based segmentation refinement of juxtavascular nodules is carried out in the following steps.

1. Based on local structure analysis, the shape features, for example, compactness or sphericity factors, are used to distinguish segmented objects in Section 2 and judge whether or not they contain the blood vessels and juxtavascular nodules. Just some filtering objects/regions need to be refined by using the following PMM-based segmentation refinement method. The correction method locally refines the nodule segmentation along recognized vessel attachments only, without modifying the nodule boundary elsewhere. This allows for computation cost to be reduced.
2. All features including 3D gradient flow direction features are extracted and computed. PMM for juxtavascular nodules is constructed. Parameters are estimated by using the generalized mixture decomposition algorithm scheme (GMDAS) of EM (expectation maximization) algorithm.
3. Classification and performance assessment.

The basic reasoning behind this algorithmic family springs from our familiar Bayesian philosophy. We assume that there are *m* clusters, *C*_*j*_,  *j* = 1,…, *m*, underlying the data set. Each vector *v*_*i*_,  *i* = 1,…, *N*, belongs to a cluster *C*_*j*_ with probability *P*(*C*_*j*_ | *v*_*i*_). A vector *v*_*i*_ is appointed to the cluster *C*_*j*_ if

(11) $$\begin{array}{c} P\left(C_{j}\;|\;v_{i}\right)>P\left(C_{k}\;|\;v_{i}\right)\;k=1,\ldots ,m,\;\;k\neq j. \end{array}$$

## 4. Classification of Pulmonary Nodules Using Knowledge-Based and C-SVM Classifier 

As mentioned above, pulmonary nodules are classified by using the knowledge-based C-SVM classifiers. First, the knowledge-based piecewise linear classification is used to remove easily dismissible nonnodule objects. Then, C-SVM classification is used to further classify nodule candidates and reduce the number of false positive (FP) objects. Here, the nodule candidates filtered by the knowledge-based piecewise linear classification are used as the training and testing samples set of C-SVM, and their intensity, shape and texture features in 2D space and 3D space, selected and extracted based on knowledge, are used as the input parameters of C-SVM. 

### 4.1. Feature Selection and Extraction Based on Knowledge

Feature selection and extraction play an important role in the intelligent recognition of pulmonary nodules. How to select and extract features is the key for intelligent detection of pulmonary nodules. In this paper, 2D and 3D features are calculated and used for detection of pulmonary nodules.

 Clinical manifestation and pathological features of pulmonary nodules in CT include position, shape, lobulation, burr sign, notch, calcification, satellite lesion, and pleural retraction sign [31, 32]. According to the pathomorphology of pulmonary nodule lesions, a small lesion appears as a circle because it has not encountered many obstacles in its growing process; when the lesion grows to a certain degree, it shows spherical shape, and lobulation and notch signs appear due to the barrier of neighborhood bronchus trees and blood vessels; as the lesion grows further, it oppresses the bronchia, blood vessel, or pleura and exhibits rough edges, meanwhile continuous burr sign and lobulation appear. Moreover, burr sign, lobulation, and cavitation with GGO edges appear blur and uneven, which are the most important pathomorphological features in mid-later growing season; thus, they can be represented by the compact ratio. Therefore, shape features are still the most important features for the detection of true pulmonary nodules. Furthermore, nodule candidate extraction is often disturbed by bronchus and blood vessels in pulmonary hilar. So, we can, therefore, select some features for detecting pulmonary nodules according to expert knowledge. For example, the area feature reflects the size of the nodule. The compactness, reflecting degree close to circle and smoothness of region, can be used to detect rough edges; the rougher the edge is, the smaller the compactness is. The concavity ratio feature reflects the degree of concavity of the region boundary. The compactness and concavity ratio features correspond to the burr sign of pulmonary nodules. The inverse difference moment feature reflects smoothness, or the degree of alternately concave and convex change in the region boundary. In summary, some features in 2D space and 3D space described in Table 1 are calculated including gray, position, shape and texture features. Suppose the size of the CT image is *M* × *N*, the gray value is *I*(*x*, *y*) at the point (*x*, *y*). In centroid calculation, *m*_*pq*_ is the *p* + *q*-order origin moment; in diameter and ellipticity calculation, *μ*_*pq*_ is the *p* + *q*-order central moment; in perimeter calculation; *n*_*e*_ is the even code number of chain code, and *n*_o_ is the odd code number; in circularity calculation, *r*_*i*_ is the radius of inscribed circle, and *r*_*c*_ is the radius of circumcircle; in slenderness and rectangle degree calculation, *W* is the width of the potential nodule object, and *H* is the height. Moreover, some features of the segmented object in 3D space are more useful to distinguish nodules and vessels, such as the volume of the segmented object and the volumetric quotient which is the ratio of the volume of the segmented object to the volume of its circumsphere whose radius is half of the long axis. Given the fact that a nodule is generally either spherical or has local spherical elements, while a blood vessel is usually cylindrical, then the volumetric quotient of the pulmonary nodule is close to 1, yet that of the vessel is far less than 1. 

### 4.2. Classifiers of Pulmonary Nodules Based on Knowledge-Based C-SVM

#### 4.2.1. Knowledge-Based Piecewise Linear Classifier in 3D Space

A knowledge-based piecewise linear classifier in 3D space is designed to remove easily dismissible nonnodule objects from pulmonary nodule candidates. The feature space of knowledge-based piecewise linear classification is formed by four features, including volume, volumetric quotient, and mean and standard deviation of intensity in 3D space.

#### 4.2.2. Nodules Classification Based on Knowledge-SVM Classifier

The C-SVM is used to solve the problem of unbalanced dataset. The nodule candidates set is a typical unbalanced dataset, in which the nodule samples are far less than the non-nodule samples; so the C-SVM is fit for the classification in an unbalanced dataset. In this paper, nodule candidates are regarded as the samples set *X* = [*s*_1_, *s*_2_,…, *s*_*N*_]^*T*^, where *N* denotes the number of total samples, and *s*_*i*_ (*i* = 1,2,…, *N*) is a row vector, representing the feature set of any sample. The nodule candidates filtered by the knowledge-based classification are used as the training samples set of C-SVM, and their intensity, shape and texture features in 2D space and 3D space are taken as the input parameters of C-SVM. The input data of C-SVM are normalized to [0, 1], and the class label of C-SVM *y*_*i*_ ∈ {+1, −1},  *i* = 1,2,…, *N* (where +1 corresponds to a nodule and −1 to a nonnodule) of the corresponding sample *x*_*i*_ is also given. Then the samples' label set is *Y* = [*y*_1_, *y*_2_,…, *y*_*N*_]^*T*^ and the dataset can be represented as (*X*, *Y*). The decision function of the C-SVM is given as [33]:

(12) $$\begin{array}{c} f\left(s\right)=\mathrm{sgn}\left[\sum_{i=1}^{l}a_{i}y_{i}K\left(s_{i},s_{j}\right)+b\right], \end{array}$$

where *K*(*s*_*i*_, *s*_*j*_) is a nonlinear kernel function, *b* ∈ *R*, and *a* is constrained as follows: 0 ≤ *a*_*i*_ ≤ *C*_+_, for *y*_*i*_ = +1, and 0 ≤ *a*_*i*_ ≤ *C*_−_, for *y*_*i*_ = −1 where *C*_+_ and *C*_−_ are penalties for class +1 and −1, respectively.

In order to increase the efficiency of the SVM, a training method is used to train the SVM, which uses the grid search method to search the optimal parameters of C-SVM and selects the sequential minimal optimization (SMO) working set, using second-order information to achieve fast convergence. Also the RBF (radical basis function) kernel function is used in this paper, defined as

(13) $$\begin{array}{c} K\left(s_{1},s_{2}\right)=\exp\left(-\gamma {||s_{1}-s_{2}||}^{2}\right), \end{array}$$

where *γ* is the parameter. 

## 5. Experimental Results

Two databases were used to evaluate the effectiveness of the proposed method: one consisting of 60 thoracic CT scans obtained from LIDC database [34], another database consisted of 60 thoracic CT scans from several hospitals. The used medical CT slices were data sets with an intensity value of 16 bits and a resolution of 512 ∗ 512. Slice thickness varied from 0.5 to 2.5 mm and the total slice number for each scan varied from 52 to 384 with an average of 135/scan. The X-ray tube current ranged from 30 to 250 mA, and the pixel size ranged from 0.5 mm/pixel to 0.7 mm/pixel. Pulmonary nodules in CT images are solid or GGO (part-solid or nonsolid), whose sizes are from 3 mm to 30 mm. Moreover, locations of nodules are uncertain; some are isolated, others are adhered to lung wall or blood vessels. Each scan was read individually by members of a qualified panel and then a consensual gold standard was defined by the panel. This process defined ground truth of 164 nodules, and the number of different kinds of nodules is shown in Table 2. 

The whole dataset was randomly split into training and testing datasets with the same number of scans (60 scans each). The latter was used as the independent testing for evaluating the performance of the trained classifiers, which has 86 nodules. Some experimental results in each step of the detection of pulmonary nodules as well as some discussions are presented below.

The whole dataset was randomly split into training and testing datasets with the same number of scans (60 scans each). The potential nodule objects for training and testing data sets of classification are generated by using the proposed segmentation method described in Sections 2 and 3. The latter was used as the independent testing for evaluating the performance of the trained classifiers, which has 86 nodules. Some experimental results in each step of the detection of pulmonary nodules as well as some discussions are presented below.

### 5.1. Segmentation of Potential Nodule Objects

The training dataset was processed, and 1377 potential nodule objects were segmented by using the proposed segmentation method described in Sections 2 and 3. 

#### 5.1.1. Qualitative Validation

In order to validate the effect of the proposed FIACM-based segmentation method and PMM-based refinement method, the clinical data with GGO nodules and juxtavascular nodules should be segmented and explored. 

**Figure 4:**
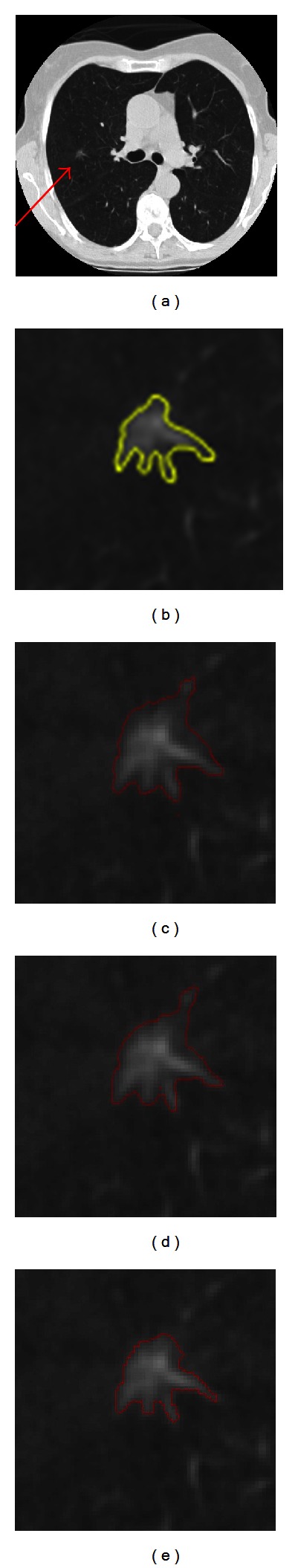
Segmentation result of a nonsolid GGO pulmonary nodule. (a) Original CT image; (b) the segmentation result carried out by experts; (c) the segmentation result by region-based active contour model; (d) the segmentation result by integrated active contour model; and (e) the segmentation result by the proposed FIACM model.

A comparison for segmentation of GGO nodule between the proposed FIACM-based segmentation method and the traditional approach, for example, region-based active contour model [7] and integrated active contour model [14], is shown in Figure 4. From Figure 4, the described segmentation method outperforms the traditional methods. As shown in Figures 4(c) and 4(d), the problem of boundary leakage at boundaries of a GGO pulmonary nodule occurs, while the problem is solved in Figure 4(e).

**Figure 5:**
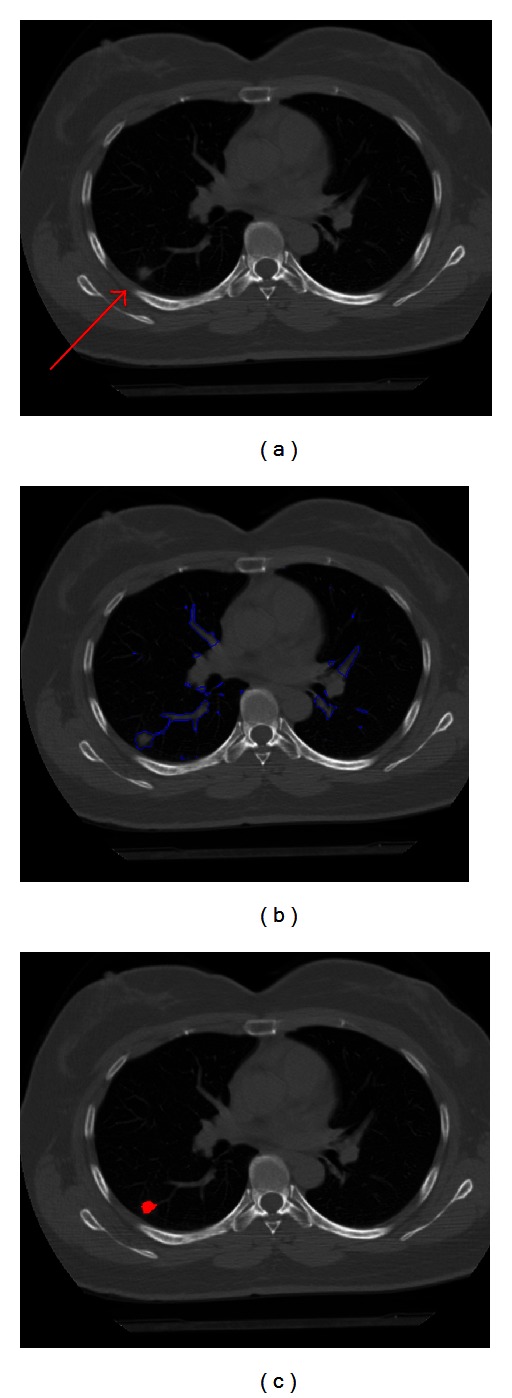
Segmentation results of the juxtavascular pulmonary nodule. (a) Original CT image, nodule adjacent to blood vessels; (b) whole segmentation result by FIACM-based method; and (c) segmented juxtavascular nodule after the fine segmentation by using PMM-based refinement method.

**Figure 6:**
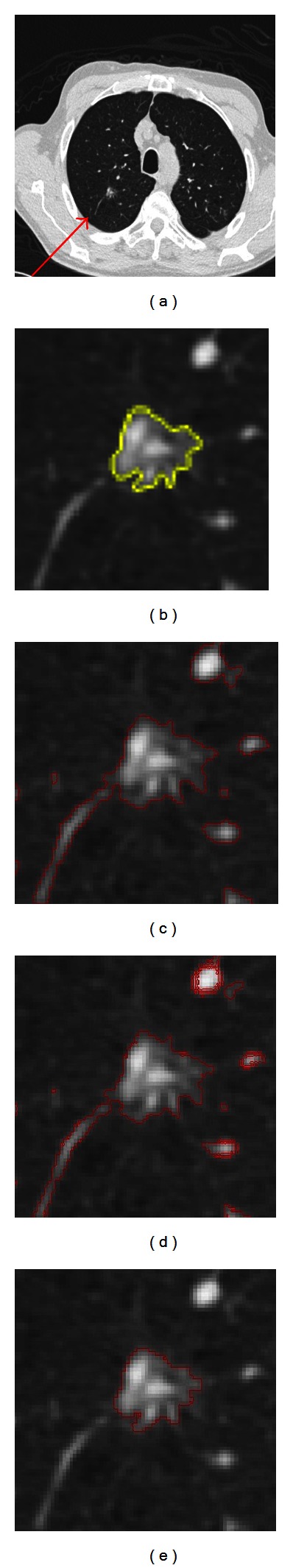
Segmentation results of the juxtavascular pulmonary nodule. (a) Original CT image; (b) the segmentation result carried out by experts; (c) the segmentation result by region-based active contour model; (d) the segmentation result by integrated active contour model; and (e) the segmentation result by the proposed FIACM-based segmentation and PMM-based refinement method.

In this paper, juxtavascular nodules are segmented under a single segmentation framework. First, it is segmented by using FIACM-based segmentation method; then refinement is achieved by using PMM-based segmentation refinement method, shown in Figure 5. The refinement procession is only applied to pixels in some regions containing the blood vessels and juxtavascular nodules. For this reason, the correction and refinement method has the advantage of locally refining the nodule segmentation along recognized vessel attachments only, without modifying the nodule boundary elsewhere. Figures 5 and 6 illustrate the segmentation results. From Figures 5 and 6, the described segmentation method outperforms the traditional methods.  Figure 6 shows the segmentation results of a juxtavascular pulmonary nodule using the proposed method, region-based active contour model [7], and the integrated active contour model [14], respectively. As shown in Figures 6(c) and 6(d), the problem of boundary leakage occurs in the adhesion place between the juxtavascular nodule and its attached vessel, while the problem of boundary leakage does not occur in Figure 6(e), which is solved by the proposed FIACM-based segmentation and PMM-based refinement method.

#### 5.1.2. Quantitative Validation

Beyond the visual inspection, a quantitative analysis is necessary to ascertain the accuracy of the proposed segmentation method. 

In this paper, the well-known Tanimoto/Jaccard error *A*(*C*_*m*_, *C*_*o*_) [12] is used as the validation merics, which refers to distances between segmentation results or to volume overlaps between the gold standard and the proposed segmentation method. The gold standard typically is a high-quality reference segmentation carried out by experts. *A*(*C*_*m*_, *C*_*o*_) is defined as

(14) $$\begin{array}{c} A\left(C_{m},C_{o}\right)=1-\frac{\int_{C_{m}\cap C_{o}}dx\;dy\;dz}{\int_{C_{m}\cup C_{o}}dx\;dy\;dz}, \end{array}$$

where *C*_*m*_ and *C*_*o*_ are the extracted and the desired contours, respectively.

In Table 3, the desired contour extracted manually and compared with the segmentation contours by the proposed method, the region-based active contour model using local region information, and the integrated active contour model combining curvature and statistical information. Table 3 shows that the errors of the proposed method are less than the other two traditional methods.

### 5.2. Training of SVM

After the preprocessing step, 1377 potential nodule objects in the training dataset were detected, including 78 pulmonary nodules and 1299 negative samples (nonnodules), shown in Table 4. 

For the training dataset, the knowledge-based piecewise linear classifier was firstly used to remove easily dismissible FP regions. Some easily dismissible nonnodule objects are removed from pulmonary nodule candidates, and by using a a knowledge-based piecewise linear classifier, in total, 395 nodule candidates were generated at the initial stage (317 nonnodule regions and 78 nodule regions). As discussed in Section 4.2.1, the four features, such as volume, volumetric quotient, and mean and standard deviation of intentisy are calculated and used for knowledge-based piecewise linear classification. After the piecewise linear filtering, for the remaining nodule candidates, the weighted C-SVM was then employed to further remove FP regions. The weighted SVM was trained using the following scheme. The nodule candidates are classified by k-cross-validation training and testing the SVM. After that, the grid search method is used to search the optimal parameters *C*_−_ and *γ* of C-SVM in this paper. In the grid search method, there should be a criterion to determine the optimal parameters. However, how to find a best criterion is still a difficult problem in the case of unbalanced dataset. Sensitivity and specificity are often used to measure the performance of the classification system; but they are often a tradeoff. The criterion of AUC (area under the ROC curve) is chosen to train C-SVM. When AUC reaches the maximum, the minimums of *C*_−_ and *γ* meeting the AUC condition will be chosen. In Table 5, when sensitivity reaches 87.5%, specificity is 96.8%, and the performance of the SVM is considered the optimum.

### 5.3. Validation of the Proposed Detection Method

The proposed detection method and trained model was tested on the independent data. Experimental results of recognition for pulmonary nodules show desirable performances of the proposed method. The experimental results using the proposed method indicate the performances with a detection rate of 95.4% with 1.1 FPs/scan (sensitivity of 88.2%), shown in Table 6.

By using the proposed method, Table 7 shows the variation in sensitivity and FP rate over all cases on the independent testing data. As it is known that different imaging parameters (e.g., different slice thickness and different tube currents) may affect the nodule detection performance, the proposed method tries to limit the influence by tuning the model (choosing the optimal parameters) on a wide range of nodules with different sizes, slice thickness, and radiation. The experimental results on the independent dataset demonstrate the generalizability of the proposed method.

**Figure 7:**
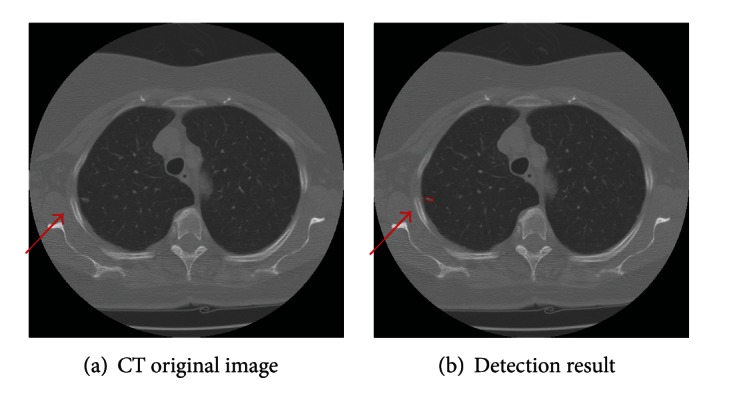
Detection result of the case containing a small GGO nodule.

**Figure 8:**
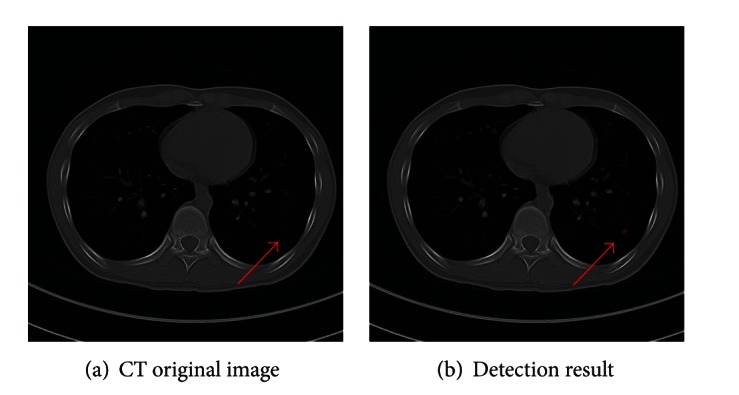
Detection result of the case containing a small pure GGO nodule.

**Figure 9:**
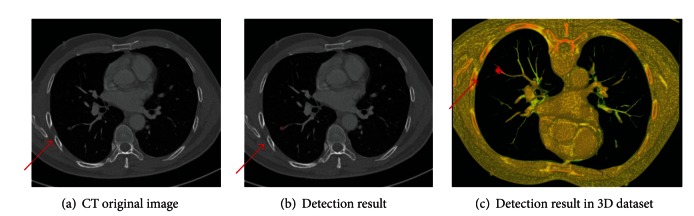
Detection results of the case containing a juxtavascular nodule.

Some detected results of the proposed method are shown in Figures 7, 8, and 9, which are the detection results of GGO nodules and juxtavascular nodules, respectively.

Table 8 illustrates the final detection sensitivity based on the different nodule size groups for the GGO nodules, juxtavascular nodules, and others, by using the proposed method on the independent testing data. Others include some solid solitary nodules and juxtapleural nodules. And Table 9 shows the detection performance for GGO and juxtavascular pulmonary nodules. Some juxtavascular nodules are nonsolid, hence they are also GGO nodules. In our experiment, there are two types of nodules, juxtavascular nodules and GGO nodules. These types are registered, respectively. 

The proposed CAD algorithm was implemented and tested on the computer with 2.13 ∗ 2 GHz CPU, 4 GB Memory and Graphic Card FX 5800. On average, it takes about 1.06 min/scan. 

## 6. Discussion

The experimental result shows a detection accuracy rate of 95.4% with 1.1 FPs/scan (sensitivity = 88.2%) for the proposed method using the independent dataset. The detection performance for GGO and juxtavascular pulmonary nodules was the detection of 96.5% with 2.7 FPs/scan, and the detection of 95.1% with 3.1 FPs/scan, respectively.

We attempt a comparison with the results reported by other research groups. Most of algorithms have been developed for solid nodules. Lee et al. [35] proposed a genetic algorithm (GA) template matching (GATM) technique for detecting nodules within the lung area. Shape and gradient features rules were used to reduce false positives (FPs). A sensitivity of 72% was achieved with 31 FP/scan. Paik et al. [5] proposed surface normal overlap (SNO) method to capture the concentration of normals by calculating derivatives of intensity images. Results on eight chest datasets were reported with 90% sensitivity and 5.6 FP/scan for solid nodules. Recently, for detection of GGO nodules, Kim et al. [36] used texture features and a three-layered neural network to detect GGO nodules. They tested 14 scans with tube dose from 200 to 400 mA and achieved a sensitivity of 94.3%. Ye et al. [4] proposed a shape-based SVM method for detecting nodules. The 3D local geometric and statistical intensity features were used to detect potential solid and GGO nodule. A detection rate of about 90.2% (including solid and GGO nodules) and FP at 8.2/scan was achieved. Unfortunately, most authors did not report quantitative results for different typologies of nodules according to proximity to surrounding structures (such as well-circumscribed, juxtavascular nodules). Bae et al. [37] used the morphologic matching algorithm to detect pulmonary nodules. An overall sensitivity of 95.1% for all nodules was achieved. The sensitivity for detecting nodules according to category was 97.4% for isolated nodules, 92.3% for juxtapleural nodules, and 94.1% for juxtavascular nodules. Diciotti et al. [3] refined the segmentation for juxtavascular nodules based on a local shape analysis of the initial segmentation making use of 3D geodesic distance map representations. They observed a percentage of successful segmentations of 84.8% in fully automated mode and of 91.0% by using an additional interactive mode (for improving the segmentation quality of juxtavascular nodules). However, nonsolid juxtavascular nodules (ground glass opacities) were not considered in their work.

Comparing with different CAD models covered in the literature [1, 4] and other reported literatures above, it seems that the proposed method's relatively high detection rate, fast computation, and applicability to different imaging conditions and nodule types show much promise for clinical applications. As a rule, nodule detection systems consist of several steps: (a) preprocessing; (b) object segmentation/candidate detection; (c) false positive reduction; and (d) classification. Most techniques often try to cheaply segment and detect the potential nodules in an attempt to drastically reduce the number of these FPs. These steps are, however, crucial in improving the detection rate and reducing the number of false positives. The reason why the proposed method has a better performance for detecting all types of GGO and juxtavascular nodules are as follows.

1. Candidate detection: the purpose of candidate detection is to avoid missing potential nodules. Moreover in this step, the precise segmentation of potential nodule is often a necessary step to computer analysis, extraction, and computation of features, which is important for false positive reduction. As mentioned before, our solution for efficiently segmenting the potential nodule objects involves two steps: (i) a FIACM-based segmentation method for a whole segmentation and (ii) a segmentation refinement method based on PMM, for accurate segmentation of potential juxtavascular nodules. The former is especially used for low-contrast nodules such as part-solid and nonsolid GGO nodules, to overcome the problems of boundary leakage, “weak” local minima and high computational cost, while the latter, referred to as a fine segmentation, is used to segment potential juxtavascular nodules. So the correction method has the advantage that it locally refines the nodule segmentation along recognized vessel attachments only, without modifying the nodule boundary elsewhere.
2. False positive reduction: in this paper, pulmonary nodules are classified by using the knowledge-based C-SVM classifiers. First, the knowledge-based piecewise linear classification is used to remove easily dismissible nonnodule objects. Then, C-SVM classification is used to further classify nodule candidates and reduce the number of false positive (FP) objects. Moreover, 2D and 3D features are used for classification of potential nodule objects.

However, some nodules (false negatives) are missed by the proposed method. Typically, these nodules are too small (almost 2 mm) and juxtapleural nodules and of very low contrast, which makes it difficult to segment and extract the effective features. 

To further improve the detection performance, some improvements need to be further investigated as follows: (a) in order to recognize small and juxtapleural pulmonary nodules in noisy image more effectively, an adaptive smoothing method needs to be further investigated, and the juxtapleural and pleural-tail nodules should be further researched; (b) in this paper, intelligent recognition of pulmonary nodules is studied. However, intelligent differentiation of benign and malignant nodules is not considered. This requires further investigation.

## 7. Conclusions

In this paper, an improved detection method of pulmonary nodules in chest CT images, combining FIACM-based segmentation method, segmentation refinement method based on PMM of juxtavascular nodules, and knowledge-based C-SVM classifier, is proposed for detecting various types of pulmonary nodules, especially for GGO nodules (part-solid and nonsolid) and juxtavascular nodules. This study demonstrates the superiority of the proposed method. The described segmentation method outperforms the traditional methods, and evaluating the algorithm on the provided test data leads to an average Tanimoto/Jaccard error of 0.11 and 0.13 for GGO and juxtavascular nodules, respectively. The experimental results using the proposed method indicate the performances with a accuracy rate of 95.4% with 1.1 FPs/scan (sensitivity of 88.2%). Different types of challenging nodules such as low-contrast part-solid/nonsolid GGO nodules and juxtavascular nodules are identified. The detection performance for GGO and juxtavascular pulmonary nodules is the detection of 96.5% with 2.7 FPs/scan and the detection of 95.1% with 3.1 FPs/scan, respectively. Experimental results of recognition for pulmonary nodules show desirable performances of the proposed method.

## Figures and Tables

**Table 1:** Features calculation of every potential nodule object.

	Features	Definition
Intensity	Mean value *G*_*m*_	*G*_*m*_ = ∑_*x*=1_^*M*^∑_*y*=1_^*N*^ *I*(*x*, *y*)/(*M* × *N*), in 2D space
	Standard deviation *G*_*u*_	*G*_*u*_ = ∑_*x*=1_^*M*^∑_*y*=1_^*N*^(*I*(*x*, *y*) − *G*_*m*_)^2^, in 2D space
	Mean *G*_*m*3D_ of intensity	In 3D space
	Standard deviation *G*_*u*3D_ of intensity	In 3D space

Position	Centroid (*i*_0_,*j*_0_)	*i*_0_ = *m*_10_/*m*_00_, *j*_0_ = *m*_01_/*m*_00_

Shape	Area *A*	*A* = ∑(*f*(*x*, *y*) = 1), in 2D space
	Perimeter *L*	$L=n_{o}+\sqrt{2}n_{e}$, in 2D space
	Diameter *a*	Long axis in 2D space, $a=2\times {\left[[2(\mu_{20}+\mu_{02}-\sqrt{{\left(\mu_{20}-\mu_{02}\right)}^{2}+4\mu_{11}^{2}})]/\mu_{00}\right]}^{1/2}$
	Ellipticity *e*	*e* = *a*/*b*, *a*, *b* are the long axis and short axis, respectively, in 2D space
	Circularity *C*	*C* = *r*_*i*_/*r*_*c*_, in 2D space
	Slenderness *S*	*S* = min⁡(*W*, *H*)/max⁡(*W*, *H*), in 2D space
	Rectangle degree *R*	*R* = *A*/(*W* × *H*), in 2D space
	Compactness *F*	*F* = 4π*A*/*P* ^2^, *P* is the perimeter of region contour. *A* is the area in 2D space Compactness ratio reflects degree closing to circle and smoothness of region. And *F* can be used to detect the rough feature of edge
	Concavity ratio *E*	*E* = *S*_*e*_/*S*, *S* is area of concave region, *S*_*e*_ is difference of convex hull and original region, and *E* reflects cupped degree of boundary
	Volume	In 3D space
	Volumetric quotient	In 3D space
	The long axis of the circumsphere	In 3D space

Texture	Energy, contrast, entropy, and adverse moment	In 2D space

**Table 2:** The number of GGO, juxtavascular, and other nodules.

Nodule type	GGO pulmonary nodule	Juxtavascular pulmonary nodule	Others	Total
Number	49	80	35	164

**Table 3:** Segmentation measure results (error rate).

CT image	The edge-based active model	The region-based active mode	The classical integrated active contour model	The proposed active contour model	
GGO pulmonary nodule	0.21	0.26	0.16	0.11	FIACM-based segmentation
Juxtavascular pulmonary nodule	0.21	0.29	0.19	0.13	FIACM-based segmentation + PMM-based refinement

**Table 4:** Experimental training data set (60 scans with 78 nodules).

Data set	Positive samples	Negative samples	Feature number
Pulmonary nodules	78	1299	21

**Table 5:** *K*-fold CV results using the grid search for optimal parameters of C-SVM classifier.

Parameters	TP	FN	TN	FP	Sensitivity	Specificity	Accuracy	AUC
*K* = 3, *C*_−_ = 0.5, and γ = 1	18	9	85	21	0.667	0.802	0.774	0.7529
*K* = 4, *C*_−_ = 4, and γ = 0.25	13	7	72	8	0.650	0.9	0.85	0.7769
*K* = 5, *C*_−_ = 4, and *γ* = 2	11	4	52	11	0.733	0.825	0.808	0.7359
*K* = 6, *C*_−_ = 2, and γ = 1	10	3	46	7	0.769	0.868	0.848	0.7688
*K* = 7, *C*_−_ = 2, and γ = 4	7	4	42	3	0.636	0.933	0.875	0.7769
*K* = 8, *C*_−_ = 2, and γ = 8	7	2	36	3	0.778	0.923	0.896	0.8610
*K* = 9, *C*_−_ = 0.5, and γ = 0.125	7	1	32	3	0.875	0.914	0.907	0.9116
*K* = 10, *C*_−_ = 8, and γ = 0.5	7	1	30	1	0.875	0.968	0.949	0.9159

**Table 6:** Detection performance of the proposed method on the testing dataset (60 scans with 86 nodules).

Total nodule	Nodule detected	Accuracy rate	FP per scan	Sensitivity
86	83	95.4%	1.1/scan	88.2%

**Table 7:** The variation of nodule detection performance over all cases on independence testing data based on the proposed method.

	Highest	Lowest	STD (standard deviation)
Sensitivity	100%	64%	0.089
Specificity	100%	81%	0.093
False positive	6	1	1.7

**Table 8:** The different nodule sizes on independent testing data.

Nodule type	≤5 mm	5–10 mm	10–20 mm	Total
GGO pulmonary nodule	3	10	16	29
Juxtavascular pulmonary nodule	2	27	12	41
Others	4	6	8	18

**Table 9:** Detection performance for GGO and juxtavascular pulmonary nodules (60 scans).

Nodule type	Total nodule	Nodule detected	Detection rate	FP per scan
GGO pulmonary nodule	29	28	96.5%	2.7/scan
Juxtavascular pulmonary nodule	41	39	95.1%	3.1/scan
